# Cost-benefit analysis of intervention policies for prevention and control of brucellosis in India

**DOI:** 10.1371/journal.pntd.0006488

**Published:** 2018-05-10

**Authors:** Balbir B. Singh, Polychronis Kostoulas, Jatinder P. S. Gill, Navneet K. Dhand

**Affiliations:** 1 School of Public Health & Zoonoses, Guru Angad Dev Veterinary and Animal Sciences University, Ludhiana, Punjab, India; 2 School of Health Sciences, University of Thessaly, Karditsa, Greece; 3 School of Veterinary Science, The University of Sydney, Camden, NSW, Australia; Yale University Yale School of Public Health, UNITED STATES

## Abstract

**Background:**

Brucellosis is endemic in the bovine population in India and causes a loss of US$ 3·4 billion to the livestock industry besides having a significant human health impact.

**Methods:**

We developed a stochastic simulation model to estimate the impact of three alternative vaccination strategies on the prevalence of *Brucella* infection in the bovine populations in India for the next two decades: (a) annual mass vaccination only for the replacement calves and (b) vaccination of both the adult and young population at the beginning of the program followed by an annual vaccination of the replacement calves and, (c) annual mass vaccination of replacements for a decade followed by a decade of a test and slaughter strategy.

**Findings:**

For all interventions, our results indicate that the prevalence of *Brucella* infection will drop below 2% in cattle and, below 3% in buffalo after 20 years of the implementation of a disease control program. For cattle, the Net Present Value (NPV) was found to be US $ 4·16 billion for intervention (a), US $ 8·31 billion for intervention (b) and, US $ 4·26 for intervention (c). For buffalo, the corresponding NPVs were US $ 8·77 billion, US $ 13·42 and, US $ 7·66, respectively. The benefit cost ratio (BCR) for the first, second and the third intervention for cattle were 7·98, 10·62 and, 3·16, respectively. Corresponding BCR estimates for buffalo were 17·81, 21·27 and, 3·79, respectively.

**Conclusion:**

These results suggest that all interventions will be cost-effective with the intervention (b), i.e. the vaccination of replacements with mass vaccination at the beginning of the program, being the most cost-effective choice. Further, sensitivity analysis revealed that all interventions will be cost-effective even at the 50% of the current prevalence estimates. The results advocate for the implementation of a disease control program for brucellosis in India.

## Author summary

Brucellosis is an endemic zoonosis in India and recent studies demonstrate that the disease results in a median loss of US$ 3.43 billion in livestock populations. Lack of resources to compensate farmers and a ban on cow slaughter means that test and slaughter policy to control brucellosis cannot be implemented in India. This is the first systematic analysis of a brucellosis control program interventions for bovine brucellosis in India. The cost-benefit analysis was successfully conducted and indicated benefits of implementing the intervention policies. For each intervention, our results indicate that the prevalence of *Brucella* infection will drop below 2% in cattle after 20 years of the implementation of disease control program although some strategies were better than others. The expected net present value (NPV) was found to range from US $ 4·16 to $ 8·31 billion for cattle and from $ 7·66 to $ 13·42 billion in buffalo for the three strategies investigated. The benefit cost ratio (BCR) ranged from 3·16 to 10·62 for cattle and from 3·79 to 21·27 for buffalo. The results advocate for the implementation of a disease control program and will help development of an official health policy for the control of brucellosis in India.

## Introduction

Brucellosis is an important zoonotic disease causing infertility, repeat breeding, retention of placenta and abortion in cattle. Humans in contact with animals usually get infected by coming in direct or indirect contact with reproductive secretions and excretions from infected animals. The disease is quite painful among humans and causes undulant fever, chills, fatigue, joint and muscle pain. If not treated, the disease can last for months and years and can cause orchitis, epididymis and endocarditis. Successful implementation of disease control programs have resulted in the eradication of brucellosis from domestic livestock in most of the developed countries [1]. However, the disease is still prevalent and classified as a neglected zoonosis in many parts of the developing world [2].

The disease is endemic in most of the production animals in India [3, 4]. With the reported disease seroprevalence of 9.3% in cattle [5] and 16·4% in buffalo populations [6], brucellosis is a serious economic concern for the cattle and buffalo industry [7]. Recent studies in India demonstrate that brucellosis in livestock populations results in a median loss of US$ 3·43 billion, with more than 95% of the losses occurring in the cattle and buffalo industry [7].

Brucellosis can be successfully controlled using appropriate intervention policies. Lack of resources to compensate farmers and a ban on cow slaughter in most parts of the country means that test and slaughter policy to control brucellosis cannot be implemented in India. There is no treatment for the disease in animals. Therefore, vaccination of cattle and buffalo population remains the sole alternative for the prevention and control of brucellosis in livestock populations in the country. However, information of benefits and costs of implementing intervention strategies to control the disease in India are largely unknown.This study aims to assess the costs and benefits of alternative control strategies for brucellosis in India. Initially, a stochastic simulation model was developed to project the course of *Brucella* infection for the national cattle and buffalo herd, over the next twenty years, under two different vaccination schemes. Subsequently, we performed a cost-benefit analysis to quantify the expected benefits of the proposed alternatives. We anticipate that this study would help policy makers to adopt the best available long-term intervention policy to prevent and control the occurrence of brucellosis in livestock and human populations in India.

## Methods

### Overall approach and alternative control strategies

Firstly, we developed a stochastic simulation model to estimate the impact of alternative vaccination strategies on the prevalence of *Brucella* infection for the cattle and buffalo populations in India, for the next two decades. The considered alternatives were based on published literature [8–10]. For the first intervention, we assumed a planned annual livestock mass vaccination campaign using *Brucella abortus* S19 for the female bovine (cattle and buffalo) replacement calves. For the second intervention, we assumed that all the adult and young female bovine populations will be vaccinated at the beginning of the program, followed by an annual vaccination of only replacements. The third intervention considered the annual mass vaccination of replacements for a decade followed by a decade of a test and slaughter strategy. We quantified the expected benefits and gains of the proposed control programs and performed a benefit-cost analysis to calculate the overall net expected benefit for each intervention.

### Modelling framework

To estimate the expected benefits from the alternative vaccination strategies, the following dynamic, synchronous, discrete time event stochastic simulation model was setup. First, animals were generated within herds. The time step (*t*) for this model was one year. At each time step, (a) the life stage (i.e. age) of each animal was determined by a dynamic component that is based on data about the age distribution and the age-specific replacement rate for the cattle and the buffalo populations; and (b) the infection stage for each animal was based on the expected prevalence of brucellosis for each species. Prevalence (*P*) was simulated at the herd level and animals within the same herd were assumed to attain the same risk of getting infected. For each herd, *P* was simulated for the first year and for each of next years it was based on the mean prevalence estimate of the previous year *P*_*t-1*_.

Animals that got infected were assumed to remain infected for life. Replacements and animals that were not infected were assumed to attain a yearly risk (*YR*_*t*_) of getting infected that depended on *P*_*t-1*_ and the expected mean duration (*D*) of the disease in the infected animals:

$${YR}_{t}=1-e^{-\frac{P_{t-1}}{(1-P_{t-1})D}}$$

The model was allowed to run for a “burn-in” phase of 50 years and then each of the alternative interventions was considered: (a) annual mass vaccination only for the replacement calves and (b) vaccination of both the adult and young population at the beginning of the program followed by an annual vaccination of the replacement calves and (c) annual mass vaccination of replacements for a decade followed by a decade of a test and slaughter strategy. Vaccination was assumed to provide complete protection from infection although we allowed for a small rate of vaccination failures and we also considered different realistic vaccination coverage rates that in real life affect vaccine efficacy. At each time step, a number of parameters was recorded among which prevalence (*P)*, replacement rate, the number of vaccinations and for the third scenario (i.e. vaccination of replacements followed by a test and cull strategy) the number of tested animals and the number of culled animals.

Estimates were based on the summaries of 4000 simulations of all animals and herds that were run for 40 times, for each species and intervention. A detailed description of the data sources and the input parameters of the model follows. Input parameters and the corresponding distributions are also summarized in Table 1 [5–7, 11–22].

**Table 1:** Parameters used for benefit-cost analysis of intervention strategies to control brucellosis in bovine populations, India (1 US $ = Rs. 60/-).

Parameter	Value	Range	Unit	Distribution	Reference
**Cattle**					
**Population**					
Total population	190904000	NA	Individual	Fixed	[11]
Total breedable female population	76685000	NA	Individual	Fixed	[11]
Number of operational holdings					
*Young*	27437200	NA	Individual	Fixed	[12]
*Adult*	44823500	NA	Individual	Fixed	[12]
*Total*	72260700	NA	Individual	Fixed	[12]
Herd size (not adjusted for mixed herds)	2·64	NA	Individual	Fixed	Calculation
Number of animals slaughtered	3192540	NA	Individual	Fixed	[11]
Slaughter rate	1·67	NA	%	Uniform (0·67–2·67)	Calculation
**Prevalence of infection**					
At inspection	9·30	NA	%	Beta (1247, 12151)	[5]
Mean calving per year per cow	1	NA	Individual	Fixed	[13]
Benefits					
Losses averted per infected animal	73·44	NA	US $	Fixed	[7]
Cost of the animal					
Calf female	50	NA	US $	Fixed	[14]
Young female	166·67	NA	US $	Fixed	[15]
Calf and Young female (after weighting)	103.50	NA	US $	Fixed	Calculation
Young male	8.33	NA	US $	Fixed	[15]
Adult female	333.33	250–416.7	US $	Uniform	[14]
Adult male	33·33	NA	US $	Fixed	Market price
Overall cost (after age and sex-wise weighting)	175.8	NA	US $	Fixed	Calculation
Average weight of the animal					
Calf female	60.14	23.39–96.89	Kg	Uniform	[16]
Young female	109.8	83.9–135.7	Kg	Uniform	[17]
Calf and young female (after weighting)	82.91	NA	Kg	Fixed	Calculation
Young male	80.25	22–138.5	Kg	Uniform	[17]
Adult female	407	264–550	Kg	Uniform	[18]
Adult male	523	364–682	Uniform	Fixed	[18]
Overall weight (after age and sex-wise weighting)	325.44	NA	Kg	Fixed	Calculation
Net loss after test and slaughter (after age and sex-wise weighting)	22.0	NA	US $	Fixed	[11], Calculation
**Buffalo**					
Population					
Total population^a^	108702000	NA	Individual	Fixed	[11]
Total breedable female population	56586000	NA	Individual	Fixed	[11]
Number of operational holdings					
Young	17678800	NA	Individual	Fixed	[12]
Adult	26312700	NA	Individual	Fixed	[12]
Total	43991500	NA	Individual	Fixed	[12]
Herd size (not adjusted for mixed herds)	2·47	NA	Individual	Fixed	Calculation
Number of animals slaughtered	9015960	NA	Individual	Fixed	[11]
Slaughter rate	8·29	NA	%	Uniform (6·3–10·3)	Calculation
**Prevalence of infection**					
At inspection	16·41	NA	%	Beta (33, 164)	[6]
Mean calving per year	1	NA	Individual	Fixed	[13]
Benefits					
Losses averted per infected animal	108·9	NA	US $	Fixed	[7]
Cost of the animal					
Calf female	83·33	NA	US $	Fixed	[14]
Young female	116.67	NA	US $	Fixed	[15]
Calf and Young female (after applying weighting)	98.01	NA	US $	Fixed	Calculation
Young male	50	NA	US $	Fixed	[15]
Adult female	583.33	416.67–750	US $	Uniform	[14]
Adult male	333·33	NA	US $	Fixed	Market price
Overall cost (after age and sex-wise weighting)	354.77	NA	US $	Fixed	Calculation
Average weight of the animal					
Calf female	48.34	24.6–72.08	Kg	Uniform	[19]
Young female	100.91	72.08–129.75	Kg	Uniform	[19]
Calf and Young female (after applying weighting)	71.49	NA	Kg	Fixed	Calculation
Young male	77.17	24.6–129.5	Kg	Uniform	[19]
Adult female	500	365–635	Kg	Uniform	[18]
Adult male	590	545–635	Kg	Uniform	[18]
Overall weight (after age and sex-wise weighting)	319.83	NA	Kg	Fixed	Calculation
Net loss after test and slaughter (after age and sex-wise weighting)	170.97	NA	US $	Fixed	[11], calculation
**Cattle and buffalo**					
Mortality risk					[20]
Calf (87/404)	21·5	NA	%	Beta (88, 318)	
Young (23/246)	9·35	NA	%	Beta (24, 224)	
Adult (60/1268)	4·73	NA	%	Beta (61, 1209)	
Male (101/483)	20·91	NA	%	Beta (102, 383)	
Female (69/1435)	4·81	NA	%	Beta (70, 1367)	
Total (170/1918)	8·86	NA	%	Beta (171, 1749)	
Prevalent case proportion in different age groups (cattle and buffalo) as compared to total prevalence					[21]
1–2 years	0·47	NA	Individual	Fixed	
2·1–3 years	0·91	NA	Individual	Fixed	
3·1–4 years	1·299	NA	Individual	Fixed	
4·1–6 years	1·61	NA	Individual	Fixed	
6·1–8 years	1·70	NA	Individual	Fixed	
>8 years	0·0	NA	Individual	Fixed	
Costings					Expert opinion
Vaccine cost	0· 0.67	NA	US $	Fixed	pers.comm.^a^
Service costs of vaccination by a paraveterinarian	0.52	NA	US $	Fixed	pers.comm.^b^
Cost of animal Identification tag and fixing costs by a paraveterinarian	0.25 + 0.52 = 0.77	NA	US $	Fixed	
Surveillance and diagnostic costs (Cost of 30 μl RBPT antigen, serum collection vial, a pair of gloves, a disposable syringe and needle, glass slide and service costs of a paraveterinarian and lab technician	0.001 + 0.0875 + 0.104 + 0.04 + 0.023 + 0.521 + 0.6075 = 1.384	NA	US $	Fixed	pers.comm.^c^
Cost of a health education pamphlet (per animal)	0·333	NA	US $	Fixed	
Overhead charges 10%	0.367	NA	US $	Fixed	
Total costings	4·0	NA	US $	Fixed	
Beef Price per Kg	1·877	NA	US $	Fixed	[22]
Dressing percentage	50	NA	%	Fixed	Expert opinion

(1 US $ = Indian Rupees 60)

^a^Dr Sikh Tejinder Singh, Associate Professor, Directorate of Livestock Farms, Guru Angad Dev Veterinary and Animal Sciences University (GADVASU), Ludhiana

^b^Dr Satinder Pal Singh, Veterinary Officer, Punjab Veterinary Vaccine Institute, Government of Punjab

^c^Professor RS Aulakh, Director, School of Public health and zoonoses, GADVASU, Ludhiana

### Sources of data and input parameters

#### Population and herd size

The official data for number of operational cattle and buffalo holdings [12] and the bovine population data were extrapolated to estimate average herd size of bovine populations in the country (Table 1). The National Sample Survey Office [12] data indicates that there are 105 921 800 total operational holdings for livestock classes. However, the sum of individual livestock holdings [12] indicated 142 420 600 individual livestock holdings in the country. Therefore, the cattle and buffalo operational holdings were adjusted to overcome the problem of mixed herds. After this, the herd size for cattle was estimated based on the national database (Table 1).

#### Age distribution

The natural life span for cattle is reported to be up to 20 years [23]. Due to the ban on cow slaughter in most parts of the country resulting in low culling rate, we developed a 20 year cycle and assumed that 95% of the cattle population to be aged from one month to fifteen years of age. As no birth control policies have been adopted in the country, we further assumed that most likely the population will be less than 1 year of age (minimum– 0 day, 5% - 1 month, mode –1 month to 1year, 95% - 15 years, and maximum 20 years). The natural life of water buffalo has been reported to be up to 29 years in captivity [24]. Due to low slaughter/culling rate, we made similar assumptions as in cattle that of a 29 year cycle assuming that 95% of the buffalo population is aged from one month to fifteen years of age. As no birth control policies have been adopted past 15 years, we further assumed that most likely population will be less than 1 year of age. (minimum– 0 day, 5% - 1 month, mode –1 month to 1year, 95% - 15 years, and maximum 29 years). For age cohort data, we assumed that for cattle and buffalo, a calf ≤ 1 year old; young stock is >1 and ≤ 3 years old; and adults are >3 years old.

#### Replacement rate

The replacement rate was estimated by summing up the mortality and culling/slaughter rate (Table 1). The mortality rate was taken from the published literature [21]. As the official male cattle population has been mentioned under young and adult populations, the combined mortality rates in calf and young populations were used in the analysis. From the sex-wise mortality data, male and female mortality rates for different age-groups were estimated (Table 1) [5–7, 11–22]. The slaughter rate for cattle and buffalo was estimated by dividing the number of animals slaughtered to the total animal populations. Due to ban on cow slaughter in most states of the country, we assumed that only culled cattle male are slaughtered. For buffalo, we assumed an equal proportion of slaughtered animals for both the sexes. Due to the absence of disease specific control programs, we assumed similar replacement rates for disease positive and negative animals.

#### Disease prevalence

The disease prevalence data for prevalence of brucellosis in cattle and buffalo was taken from the published literature. For cattle we assumed a within herd prevalence of 9·30% [5] which corresponds a *Be*(1247, 12151) and for buffalo, a within herd prevalence of 16·41% [6] which corresponds a *Be*(33,164) (Table 1).

#### Vaccine efficacy and vaccination coverage

Existing vaccine efficacy estimates range between 50% and 80% [25] and account for imperfect vaccination coverage. In this model we assumed vaccination coverage of 80%. We allowed for approximately 1% vaccination failure, *Be*(10,1000).

#### Test and slaughter

In the third scenario, we investigated the application of ‘test and slaughter’ policy for positive reactors for rapid eradication of the disease from bovine populations. The costs included conducting diagnostic costs (Table 1). The sensitivity and specificity of the diagnostic tests, i.e. Rose Bengal Plate test was assumed to be 54·9% and 97·7%, respectively [26]. For estimating slaughter price of these animals, we took into account the average body weight (Table 1) and assumed a dressing percentage of 50%. The beef carcass price was sourced from the published literature [22]. The average costs and body weights for cattle and buffalo were estimated after applying weighting for the proportion of animals in different sex and age categories (Table 1). We further assumed a 20% reduction in slaughter price of these animals because of their disease positive status. We subtracted the slaughter price from the current value of these animals to estimate the costs or losses associated with the cull and replacement of these animals (Table 1).

For intervention costs, the current market price of the *Brucella abortus* Strain 19 and the animal identification tags (pers. comm. Dr Sikh Tejinder Singh, Associate Professor, Directorate of Livestock Farms, GADVASU, Ludhiana) were used (Table 1). The vaccination and animal identification services are primarily provided by the para-veterinary staff in India. We assumed that both these activities will require approximately 10 minutes each of a para-veterinarian (pers. comm. Dr Satinder Pal Singh, Veterinary Officer, Punjab Veterinary Vaccine Institute, Government of Punjab). For the surveillance and diagnostic costs, the current market price of the Rose Bengal Plate test antigen, a disposable syringe and needle, a pair of gloves, a serum collection vial and a glass slide (pers. comm. Professor RS Aulakh, Director, School of Public health and zoonoses, GADVASU, Ludhiana) were considered along with a 10 minute time cost of a para-veterinarian and a lab technician for blood collection and sample testing, respectively (Table 1). The average monthly salary of a para-veterinarian (US $ 500) and of a lab technician (US $ 583.33) working in the Punjab state of India were used to estimate the time costs for these activities (personal communication Dr Satinder Pal Singh, Veterinary Officer, Punjab Veterinary Vaccine Institute, Government of Punjab) assuming 40 working hours per week. Lastly, 10% overhead charges were added to account for other infrastructure and administrative costs. However, we did not account for a farmer’s time for being involved in these activities.

### Sensitivity analysis

For each intervention we evaluated the effect of varying input parameters on the expected benefits. Specifically, we assessed the impact of (a) reducing the initial prevalence of *Brucella* infection by fifty percent, (b) reducing the vaccination coverage to 50% and (c) having herds that are consistently unvaccinated (i.e. herds that were more likely to remain unvaccinated the next year). We also assessed the additional benefits of expanding the intervention strategies beyond the twenty year period.

### Benefit-cost analysis

The interventions were considered for *t* = 20 years and at a discount rate (*r*) of 5%. Initially, we predicted the annual costs (*C*_*t*_) and benefits (*B*_*t*_) for each strategy and subsequently calculated the net present value (NPV) by applying the discount rate:

$$NPV=\sum_{t=1}^{T}\frac{B_{t}-C_{t}}{{\left(1+r\right)}^{t}}$$

Further, for each intervention the benefit cost ratio (*BCR*) was estimated as the discounted value of the incremental benefits divided by the discounted value of the incremental costs:

$$BCR=\frac{\sum_{t=1}^{T}\frac{B_{t}}{{\left(1+r\right)}^{t}}}{\sum_{t=1}^{T}\frac{C_{t}}{{\left(1+r\right)}^{t}}}$$

The costs included vaccine costs, service costs of vaccination (transportation, cold chain, and veterinarian fee), animal identification costs (ear tagging), service costs for surveillance and diagnostics, and costs for health education program (Table 1).

The averted losses were considered as benefits for implementing the control programs [27]. Based on our previous study [7], the losses occurring due to brucellosis per infected animal were estimated by dividing total losses for each species with the number of infected animals for that species (Table 1). Due to lack of data, the health and economic burden of human brucellosis could not be accounted into the overall benefits of the control programs.

### Statistical software

The analyses were conducted using R-statistical program (R statistical package version 2.12.0, R Development Core Team, http://www.r-project.org) and we run Monte Carlo simulations for 10,000 iterations so as to determine confidence limits for these estimates.

## Results

### Prevalence reduction

**Fig 1:**
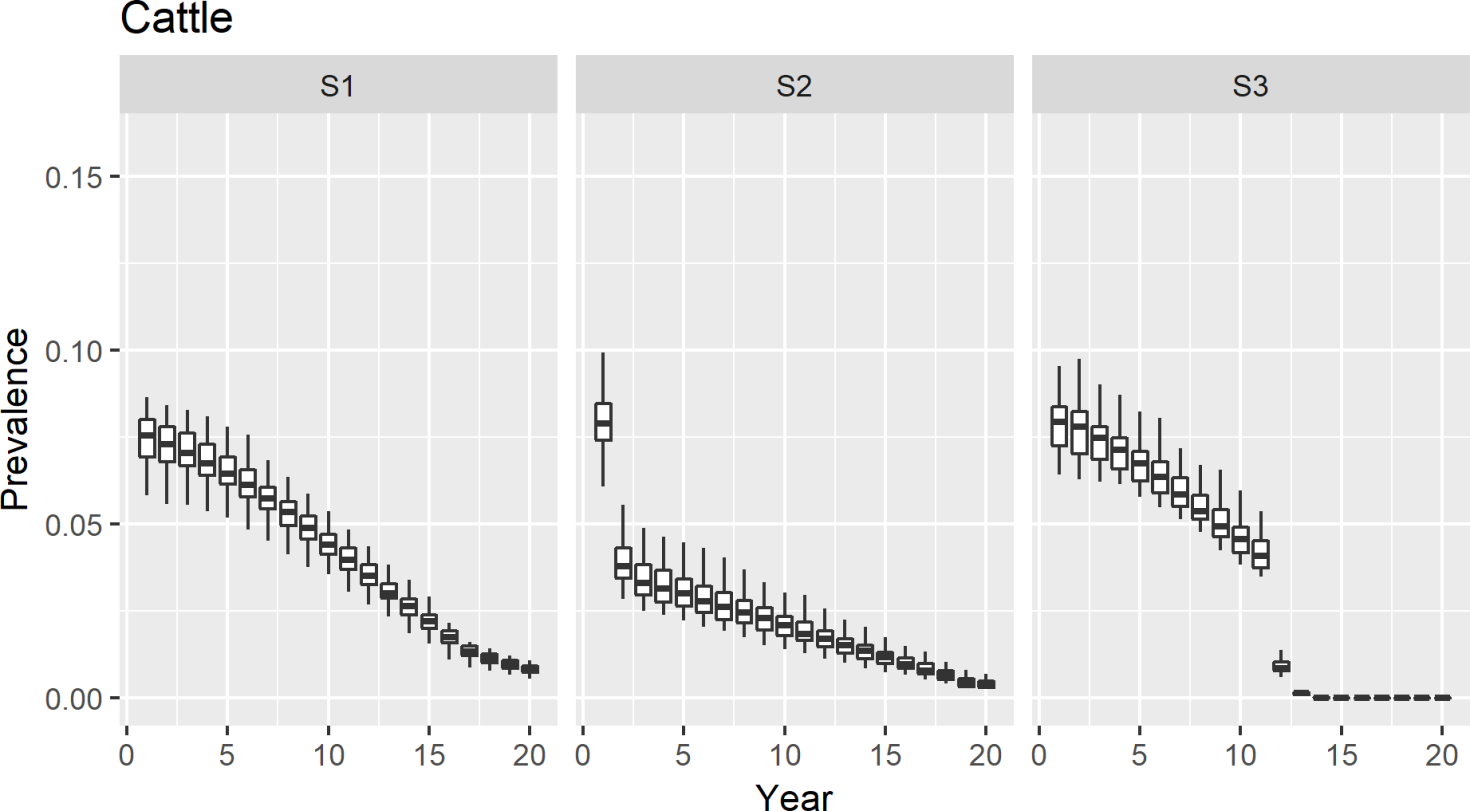
Changes in prevalence of brucellosis in cattle after the implementation of intervention programmes.

**Fig 2:**
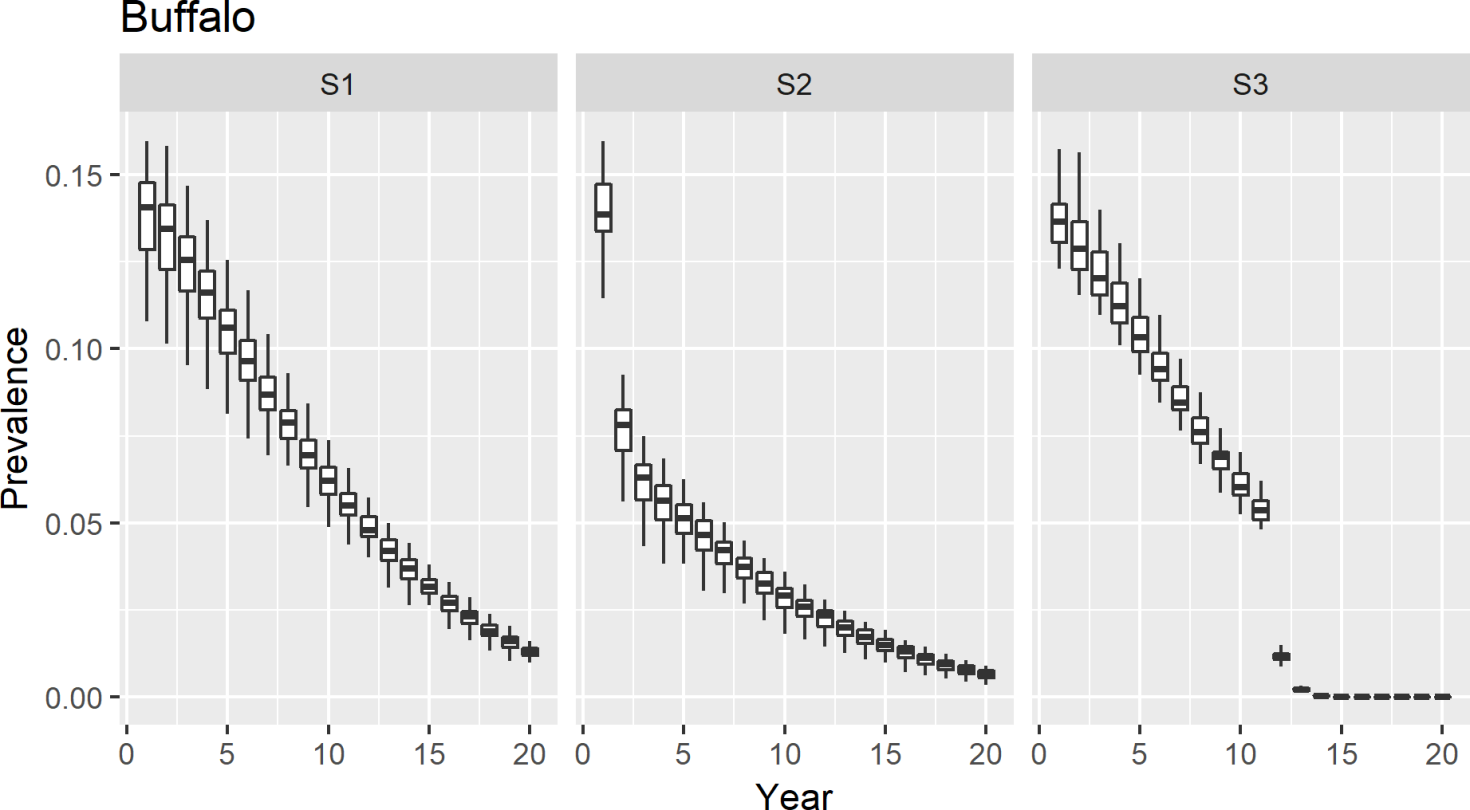
Changes in prevalence of brucellosis in buffaloes after the implementation of intervention programmes.

For each intervention, our results indicate that the prevalence of *Brucella* infection will drop below 2% in cattle after 20 years of the implementation of disease control program (Fig 1) for the cattle population. For buffaloes, a similar trend was observed. However, due to the higher initial prevalence of infection, it only drops below 3% after the twenty year implementation of all interventions (Fig 2).

### Benefit-cost analysis

The NPV during the first 20 years of the program for cattle for scenario 1, 2 and, 3 are presented in Table 2. For cattle, the NPV was found to be US $ 4·16 billion (95% CI: US $ 3·16; 5·39 billion) for the scenario 1, US $ 8·31 (6·40; 9·87) billion for the scenario 2 and, US $ 4·26 (3·26; 5·61) for the third scenario (Table 2). The results indicate that first 20 years of the programme will be cost-effective for all scenarios with the second intervention (vaccination of replacements with mass vaccination at the beginning of the program) being a significantly more cost-effective choice. The BCR for the first, second and the third intervention for cattle were 7·98 (6·29; 10·09), 10·62 (8·33; 12·5) and, 3·16 (2·66; 3·83), respectively. Similar results were obtained for buffaloes (Table 3).

**Table 2:** Net present value (NPV) and benefit-cost ratio (BCR) of a brucellosis intervention program in cattle (first 20 years) in India. Scenario 1 –Vaccination of replacements; Scenario 2 –Vaccination for all at once followed by vaccination of replacements; Scenario 3 –Vaccination of replacements for the first 10 years followed by the test and cull for remaining 10 years.

	Scenario 1 (US $) (in millions)		Scenario 2 (US $) (in millions)		Scenario 3 (US $) (in millions)	
Year	Mean	2·5^th^– 97·5^th^ percentile	Mean	2·5^th^– 97·5^th^ percentile	Mean	2·5^th^– 97·5^th^ percentile
1	-45·38	-49·05–-43·12	-314·50	-329·27–-303·791	-45·72	-47·72–-43·56
2	-17·20	-37·90–-3·78	470·34	363·14–581·41	-18·72	-49·30–7·29
3	6·90	-21·82–35·78	502·58	387·98–611·50	13·94	-15·90–51·90
4	41·06	11·38–81·89	500·91	386·43–602·19	48·70	3·51–99·95
5	72·30	36·02–117·34	495·83	385·75–595·79	87·47	38·48–150·96
6	106·71	59·81–163·67	492·13	386·21–586·16	120·84	73·71–183·65
7	144·31	92·30–203·35	485·87	379·03–570·37	158·58	104·53–230·65
8	178·91	124·51–246·12	480·26	377·49–558·12	192·07	136·48–269·34
9	209·09	150·47–279·01	474·76	377·07–549·14	222·71	168·67–294·78
10	236·52	166·37–309·36	470·50	373·29–549·21	254·21	192·65–329·31
11	262·59	201·22–337·43	462·71	363·69–538·86	281·22	223·21–348·36
12	284·27	221·81–359·60	455·66	358·98–535·62	300·89	233·63–426·47
13	304·56	238·64–386·14	449·44	350·01–530·66	373·14	288·13–486·26
14	321·38	257·25–401·43	441·06	344·72–523·53	374·12	292·81–489·99
15	334·74	268·06–415·67	431·80	337·19–513·43	357·28	280·44–461·59
16	346·52	280·58–430·92	422·77	329·96–497·24	341·14	262·87–454·76
17	354·68	284·27–434·44	414·05	322·53–492·53	325·17	250·76–422·02
18	350·25	276·01–428·99	404·69	313·24–482·80	308·57	238·35–400·76
19	342·676	268·40–418·81	395·02	305·74–471·65	294·03	232·18–382·03
20	334·10	262·49–405·30	378·70	292·97–452·25	280·12	214·83–368·46
**NPV**	4169·05	3165·07–5394·13	8314·65	6404·75–9875·19	4269·82	3266·04–5617·48
**BCR**	7·98	6·29–10·09	10·62	8·33–12·5	3·16	2·66–3·83

**Table 3:** Net present value (NPV) and benefit-cost ratio (BCR) of a brucellosis intervention program in buffalo (first 20 years) in India. Scenario 1 –Vaccination of replacements; Scenario 2 –Vaccination for all at once followed by vaccination of replacements; Scenario 3 –Vaccination of replacements for the first 10 years followed by the test and cull for remaining 10 years.

	Scenario 1 (US $) (in millions)		Scenario 2 (US $) (in millions)		Scenario 3(US $) (in millions)	
Year	Mean	2·5^th^– 97·5^th^ percentile	Mean	2·5^th^– 97·5^th^ percentile	Mean	2·5^th^– 97·5^th^ percentile
1	-40·09	-41·77–-38·23	-178·44	-184·29–-169·11	-39·95	-42·47–-37·93
2	44·96	23·78–80·17	639·84	525·20–736·31	40·02	6·99–68·02
3	128·00	91·54–183·33	752·31	637·71–856·00	119·36	68·73–151·95
4	210·97	156·21–275·16	774·13	659·84–877·19	200·22	150·73–245·52
5	290·92	213·04–363·31	785·09	673·27–886·64	273·52	223·92–336·36
6	358·14	264·56–440·13	791·97	686·22–896·45	343·41	278·17–418·93
7	421·14	320·02–519·36	793·46	684·42–901·68	404·18	328·63–482·54
8	472·13	362·96–570·98	790·32	681·54–893·20	459·55	384·18–549·56
9	515·30	399·10–619·74	785·79	670·90–889·20	500·18	415·74–602·61
10	545·80	429·87–650·71	774·93	657·29–872·46	530·58	448·97–626·08
11	569·51	446·39–680·90	761·02	645·04–864·44	554·97	478·87–661·00
12	585·61	461·17–692·32	744·14	625·61–845·34	311·51	223·55–418·64
13	595·12	466·01–705·25	725·98	610·24–823·57	537·23	441·59–678·55
14	598·96	470·09–709·30	705·90	589·79–802·29	560·01	466·29–682·42
15	599·44	471·97–708·30	684·71	570·76–784·09	538·93	466·71–671·20
16	595·29	466·51–703·94	663·19	545·90–759·08	512·33	434·08–626·04
17	587·61	461·78–696·08	640·86	527·49–733·85	488·51	415·24–603·21
18	578·48	449·10–685·31	619·06	506·78–709·99	463·72	391·97–576·42
19	566·23	440·06–669·04	596·24	488·02–682·85	445·14	382·55–554·53
20	552·37	430·74–650·99	573·44	468·45–656·00	425·82	363·37–525·77
**NPV**	8775·95	6781·75–10521·75	13424·03	11400·86–15290·25	7669·32	6493·44–9355·85
**BCR**	17·.81	14·15–21·02	21·27	18·24–24·31	3.79	3·37–4·40

### Sensitivity analysis

The NPV for the 50% prevalence estimates during the first 20 years of the program for cattle for scenario 1, 2 and, 3 are presented in S1 Table. For cattle, NPV was found to be US $ 1·78 billion (95% CI: US $ 1·07; 2·79 billion) for the scenario 1, US $ 3·27 (2·18; 4·23) billion for scenario 2 and, US $ 0·87 (-0·24; 1·71) for scenario 3 (S1 Table). For buffaloes, the NPV for the 50% prevalence estimates was found to be US $ 3·69 billion (95% CI: US $ 2·74; 4·54 billion) for the scenario 1, US $ 5·96 (4·40; 7·27) billion for the scenario 2 and, US $ 2·09 (1·02; 3·20) for the third scenario (S2 Table).

Further, sensitivity analysis revealed that for either species, disease prevalence will further reduce to less than 1% after 50 years of implementation for either intervention and will virtually lead to eradication of the disease after 100 years of the implementation programme. The long time to eradicate infection is based on the fact that we only considered realistic vaccination coverage rates. Our primary analysis, assumed a vaccination coverage of 70%. Reduction of the vaccination rate led to reduced NPV and BCR values. The same impact had the assumption that herds that were not covered were more likely to remain uncovered the next year.

## Discussion

This is the first systematic analysis of a brucellosis control program interventions for bovine brucellosis in India. Bovine brucellosis is highly prevalent in India and causes significant losses to the livestock industries. The results suggest that all of the three approaches investigated for controlling the disease would be beneficial as the prevalence of *Brucella* infection will drop below 2% in cattle and 3% in buffalo after 20 years of the implementation of disease control program. All programs had positive NPVs and >1 BCRs indicating the benefits from all programs are higher than their respective costs. The best BCR was obtained in the second intervention, i.e. vaccination of both the adult and young population at the beginning of the program followed by an annual vaccination of the replacement calves. It leads to a significant drop in prevalence at the beginning of the program and hence the risk of transmitting the disease in the subsequent years is lower. This is thus the most cost-effective approach for control of brucellosis in India. Overall, the results advocate the implementation of a disease control program for brucellosis in India.

The results of sensitivity analyses indicated that a positive effect for all interventions and a net benefit of billions of dollars for any intervention remains even after considering significantly reduced initial prevalence and vaccination coverage. This suggests that the control program would be beneficial even if some of the assumptions used in the model are changed, further supporting the implementation of a control program for the disease.

It must be noted that we only considered economic benefits of the control programs for the livestock populations. The benefits such as disability-adjusted life years (DALYs) and social losses averted due to the control programs could not be accounted. Similarly, the extra costs due to increased livestock numbers (feed costs), or unintentional consequences (abortion due to vaccinating a female cow) were not estimated. However, we believe that this will not have a major impact on the results of the current study.

Although the third approach–i.e. annual mass vaccination of replacements for a decade followed by a decade of a test and slaughter strategy–was also found to be cost-effective, it is less likely to be adopted in India because it is a Hindu majority country and Hindus consider cows to be sacred. As a result, cow slaughter is banned in most states of India. Thus it would be difficult to get community support for a strategy involving animal slaughter although it drastically reduces prevalence of the disease if implemented after a decade of vaccination. It will also be more expensive as it would involve testing of animals which would include sample collection, transport and laboratory testing. Also, there would be additional costs involved for culling infected animals. Therefore, this may not be the preferred strategy in the Indian situation. Moreover, in calculating losses for test and slaughter, we assumed that animals will be consumed after slaughter in accordance with the WHO guidelines [28]. However, it may not be feasible to do so or may increase the risk of spread of infection. Therefore, it would be more sensible to adopt a ‘test and euthanasia’ strategy in which the infected animals are euthanized and their carcasses burnt or buried and not consumed. This strategy is likely to have a greater acceptance among the community which is very essential for the success of any control program. However, this would increase the cost of the test and slaughter program as it will result in a complete loss of slaughtered animal instead of just a loss of 20% considered in the scenario. Thus the actual cost of the third scenario may be higher than we estimated.

In this study, BCRs for three inventions for cattle were estimated to range from 3·16 to 10·62 and for buffaloes from 3·79 to 21·27. Similar estimates have been obtained in some other studies conducted around the world. The strategy of vaccinating 3–6 month old female bovine, male ovine and female ovine followed by compulsory slaughter after attaining the target prevalence have been advocated in Turkey [10], where BCR was estimated to be 2·26 [10]. A BCR of 3·2 has been estimated for control of brucellosis in Nigeria [29]. A national serological survey and risk based vaccination using S19 and an awareness program was found to have a BCR of 6·8 for control of brucellosis in Nepal [9].

Note that we only considered scenarios for control of the disease; eradication was not considered feasible in the current circumstances. The disease is highly prevalent and endemic in India; therefore, it would be unrealistic to achieve eradication. Further, India is a vast country with significant movement and intermixing of animals. Moreover, eradication would definitely require test and slaughter but religious and cultural beliefs would impede implementation of any such program due to limited community support. However, once the prevalence reduces below 2% after 20 years, there may be greater community support for eradication as well as test and slaughter/euthanasia as discussed before. Therefore, it would be wise to revisit this question sometime in the future.

In this work realistic inputs of vaccination coverage aimed to also adjust for the reduced vaccine efficacy due to vaccine failure as well as problems associated with cold chains, which were not directly accounted for. Assumed vaccination rates ranged from 70% to 50% to cover different vaccine efficacies that have been used in previous studies [25, 30]. Undoubtedly, real-time data of vaccine efficacy could further improve the predictive ability of our model. However, in our sensitivity analysis we considered the realistic fact that herds that were not covered once were more likely to remain uncovered due to issues associated with inability to reach them or farmers’ will to cooperate. However, even better NPV and BCR will be achieved if the vaccination coverage/efficacy is improved.

It has been reported that 53.6% of the bovine (cattle and buffalo) population receives foot and mouth disease (FMD) vaccination in India [31, 32]. Therefore, our assumption of 50% vaccination coverage is quite realistic. However, there will be pockets of low (<10%) and high coverage (90%+) areas. Many factors such as poor infrastructure, lack of knowledge and veterinary personnel availability are responsible for poor adoption of vaccines in India [33]. A farmer’s perceptions such that vaccination could lead to decrease in milk yield, swelling and fever also decrease vaccine coverage [33]. Low community acceptance, vaccine stock outs at the local level and timeliness of vaccine also affect the vaccine coverage [34]. These factors could affect the benefits but could not be accounted in the current study.

The advantage of using the S19 vaccine is that immunity induced is long-lasting and has been reported to be effective till fifth pregnancy [10, 35]. However, there are a number of concerns with using this vaccine. The major concern is the common occurrence of the needle stick injuries and the accidental inoculation in veterinary personnel while participating in *Brucella* vaccination programs [36]. The rates of accidental exposure ranging from 6·7% to 46% have been reported [37]. The needle stick injuries have been reported to cause a low virulence human brucellosis [38]. To avoid needle stick injuries, research should be conducted in the use of a safety vaccinator as used for other vaccines such as Gudair® vaccination in Australia [39] and animals should be properly restrained before vaccination.

Additionally, the S19 vaccine could interfere with the recommended diagnostic tests and may cause abortion in the pregnant animals [35, 40]. Moreover, the vaccine cannot be used for male [41] or infected animals [42]. Therefore, there is a need for the development of a better vaccine that can differentiate infected from vaccinated animals (DIVA). RB51 vaccine can be used instead of S19 as it allows serological differentiation between naturally infected and vaccinated animals but it is not currently available in India and is considered to have a lower efficacy than S19.

It is worth mentioning here that the cost and benefit analyses evaluated in this manuscript only pertain to the effect of the disease on the domestic animal population. The benefits to the human population would be over and above the benefits discussed here but were beyond the scope of this study. It is well known that humans get infected while handling infected animals. Therefore, various studies have shown that the disease is prevalent among occupational groups such as veterinary personnel, laboratory workers, livestock farmers and abattoir workers in India [43–45]. We have recently shown that the disease causes a loss of 177 601 (95% UI 152 695–214 764) DALYs at the rate of 0.15 (95% UI 0.13–0.17) DALYs per thousand persons every year [46] and an annual median loss of Rs 627.5 million (US $ 10.46 million) in India [46]. Complete eradication of the disease will save these losses but further studies are required to investigate the real impact of the control strategies discussed in this manuscript on the human population.

## Supporting information

S1 Table Sensitivity analysis of net present value (NPV) and benefit-cost ratio (BCR) of a brucellosis intervention program in cattle (at 50% prevalence -first 20 years) in India. Scenario 1 –Vaccination of replacements; Scenario 2 –Vaccination for all at once followed by vaccination of replacements; Scenario 3 –Vaccination of replacements for the first 10 years followed by the test and cull for remaining 10 years.
(DOCX)

S2 Table Sensitivity analysis of net present value (NPV) and benefit-cost ratio (BCR) of a brucellosis intervention program in buffalo (at 50% prevalence -first 20 years) in India. Scenario 1 –Vaccination of replacements; Scenario 2 –Vaccination for all at once followed by vaccination of replacements; Scenario 3 –Vaccination of replacements for the first 10 years followed by the test and cull for remaining 10 years.
(DOCX)
