# Supplementary material for: Cost-benefit analysis of intervention policies for prevention and control of brucellosis in India
Source: PLoS Negl Trop Dis. 2018 May 10;12(5):e0006488. doi: 10.1371/journal.pntd.0006488 (PMC5963803; doi:10.1371/journal.pntd.0006488)
Supplement: S1 Table — Scenario 1 –Vaccination of replacements; Scenario 2 –Vaccination for all at once followed by vaccination of replacements; Scenario 3 –Vaccination of replacements for the first 10 years followed by the test and cull for remaining 10 years. (DOCX) [file pntd.0006488.s001.docx]

|  | **Scenario 1 (US $) (in millions)** | | **Scenario 2 (US $) (in millions)** | | **Scenario 3 (US $) (in millions)** | |
| --- | --- | --- | --- | --- | --- | --- |
| **Year** | **Mean** | **2·5^th^ – 97·5^th^ percentile** | **Mean** | **2·5^th^ – 97·5^th^ percentile** | **Mean** | **2·5^th^ – 97·5^th^ percentile** |
| 1 | -45·50 | -49·17–-42·25 | -311·76 | -324·73–-293·45 | -45·20 | -48·22–-41·71 |
| 2 | -31·94 | -47·29–-11·96 | 201·50 | 123·15–260·04 | -31·30 | -44·72–-17·67 |
| 3 | -15·11 | -32·84–13·72 | 211·91 | 142·84–269·76 | -18·29 | -41·42–2·35 |
| 4 | 2·37 | -24·36–37·37 | 210·97 | 141·90–273·32 | -3·88 | -29·09–24·19 |
| 5 | 19·49 | -13·83–52·81 | 208·60 | 139·75–270·20 | 15·32 | -21·49–56·10 |
| 6 | 37·52 | 2·44–73·30 | 206·29 | 143·27–266·63 | 35·53 | -2·77–78·27 |
| 7 | 55·65 | 22·16–93·62 | 204·49 | 140·14–262·29 | 52·21 | 12·86–94·55 |
| 8 | 73·33 | 34·33–114·41 | 201·61 | 142·85–261·31 | 69·28 | 29·83–104·66 |
| 9 | 89·78 | 48·29–134·39 | 198·44 | 140·25–258·25 | 85·08 | 37·99–122·73 |
| 10 | 104·30 | 64·71–155·76 | 195·97 | 137·73–247·82 | 99·92 | 47·95–140·15 |
| 11 | 118·96 | 76·96–176·91 | 192·24 | 133·58–240·33 | 112·55 | 54·29–156·35 |
| 12 | 130·63 | 84·64–184·96 | 188·42 | 127·77–239·13 | 24·28 | -67·66–101·83 |
| 13 | 140·36 | 92·11–195·66 | 185·36 | 124·52–239·18 | 63·39 | -31·50–150·68 |
| 14 | 148·28 | 99·66–216·28 | 181·47 | 120·91–235·79 | 67·11 | -31·64–160·12 |
| 15 | 154·48 | 105·26–225·15 | 177·81 | 118·03–231·29 | 65·45 | -19·92–142·28 |
| 16 | 159·51 | 110·66–234·81 | 173·88 | 113·99–226·01 | 62·04 | -24·29–135·31 |
| 17 | 163·73 | 112·73–245·42 | 169·96 | 111·81–219·23 | 58·78 | -25·44–131·12 |
| 18 | 161·26 | 110·92–243·28 | 165·80 | 110·12–214·77 | 56·42 | -20·01–121·87 |
| 19 | 158·29 | 108·31–237·10 | 161·34 | 107·09–212·75 | 53·57 | -20·90–120·61 |
| 20 | 154·60 | 106·73–230·96 | 154·58 | 101·40–204·19 | 51·60 | -16·54–113·22 |
| **NPV** | **1780**·**07** | **1073**·**85**–**2797**·**12** | **3278**·**98** | **2183**·**65**–**4236**·**79** | **873**·**91** | **-244**·**96**–**1715**·**74** |
| **BCR** | **3**·**98** | **2**·**77**–**5**·**69** | **4**·**80** | **3**·**50**–**5**·**93** | **1**·**45** | **0**·**87**–**1**·**88** |
