# Supplementary material for: Cost-benefit analysis of intervention policies for prevention and control of brucellosis in India
Source: PLoS Negl Trop Dis. 2018 May 10;12(5):e0006488. doi: 10.1371/journal.pntd.0006488 (PMC5963803; doi:10.1371/journal.pntd.0006488)
Supplement: S2 Table — Scenario 1 –Vaccination of replacements; Scenario 2 –Vaccination for all at once followed by vaccination of replacements; Scenario 3 –Vaccination of replacements for the first 10 years followed by the test and cull for remaining 10 years. (DOCX) [file pntd.0006488.s002.docx]

|  | **Scenario 1 (US $) (in millions)** | | **Scenario 2 (US $) (in millions)** | | **Scenario 3(US $) (in millions)** | |
| --- | --- | --- | --- | --- | --- | --- |
| **Year** | **Mean** | **2·5^th^ – 97·5^th^ percentile** | **Mean** | **2·5^th^ – 97·5^th^ percentile** | **Mean** | **2·5^th^ – 97·5^th^ percentile** |
| 1 | -39·34 | -42·06–-36·40 | -178·00 | -184·44–-171·34 | -40·30 | -42·67–-38·08 |
| 2 | -3·71 | -22·36–17·38 | 319·57 | 242·52–399·32 | -4·65 | -23·84–16·35 |
| 3 | 34·03 | 2·56–60·16 | 357·00 | 279·04–431·84 | 32·63 | 11·20–63·19 |
| 4 | 73·04 | 32·97–108·31 | 363·65 | 283·57–439·20 | 70·43 | 40·99–109·66 |
| 5 | 107·58 | 55·77–144·95 | 364·90 | 286·89–440·74 | 104·60 | 67·72–145·99 |
| 6 | 141·30 | 78·98–182·56 | 364·77 | 284·84–439·05 | 136·45 | 91·55–183·98 |
| 7 | 171·26 | 109·00–213·04 | 362·27 | 278·92–436·53 | 165·39 | 113·58–220·43 |
| 8 | 195·62 | 130·88–244·67 | 358·46 | 273·24–432·74 | 187·91 | 131·87–254·56 |
| 9 | 217·97 | 159·16–275·02 | 352·99 | 264·11–434·24 | 207·73 | 144·54–272·97 |
| 10 | 235·49 | 176·19–290·96 | 345·95 | 257·33–423·76 | 223·45 | 156·54–295·43 |
| 11 | 246·02 | 184·79–304·37 | 337·83 | 247·66–418·30 | 235·26 | 166·78–312·90 |
| 12 | 254·37 | 195·03–310·79 | 329·05 | 238·96–408·41 | -4·66 | -67·63–60·82 |
| 13 | 259·26 | 199·45–317·15 | 320·90 | 232·40–396·34 | 98·04 | 6·03–175·57 |
| 14 | 262·98 | 204·51–320·57 | 311·47 | 223·39–386·03 | 107·95 | 34·20–200·35 |
| 15 | 263·74 | 209·99–321·88 | 301·70 | 216·96–372·54 | 108·48 | 12·89–183·03 |
| 16 | 262·95 | 208·04–318·85 | 290·93 | 208·46–359·54 | 100·04 | 16·04–170·06 |
| 17 | 260·67 | 206·52–314·44 | 281·10 | 202·10–348·98 | 97·17 | 12·52–173·48 |
| 18 | 256·43 | 203·12–311·12 | 271·38 | 196·68–335·95 | 92·97 | 6·98–157·08 |
| 19 | 251·74 | 201·77–306·52 | 260·85 | 187·80–322·16 | 88·49 | 21·46–147·38 |
| 20 | 245·54 | 197·60–297·57 | 250·50 | 178·93–310·75 | 85·93 | 13·04–148·34 |
| **NPV** | **3697**·**02** | **2743**·**31**–**4544**·**94** | **5967**·**36** | **4404**·**21**–**7273**·**84** | **2093**·**37** | **1028**·**41**–**3200**·**81** |
| **BCR** | **8**·**08** | **6**·**26**–**9**·**76** | **10**·**02** | **7.74**–**11.95** | **1.81** | **1**·**40**–**2**·**22** |
